# Supplementary material for: A widespread hydrogenase supports fermentative growth of gut bacteria in healthy people
Source: Nat Microbiol. 2025 Oct 23;10(11):2686–701. doi: 10.1038/s41564-025-02154-w (PMC12578642; doi:10.1038/s41564-025-02154-w)

nature microbiology

Supplementary information

https://doi.org/10.1038/s41564-025-02154-w

A widespread hydrogenase supports fermentative growth of gut bacteria in healthy people

In the format provided by the authors and unedited

Supplementary information

A widespread hydrogenase supports fermentative growth of gut bacteria in healthy people

Supplementary notes

Supplementary Note 1. Hydrogenase expression and activity in Clostridia and Actinobacteria. Patterns of hydrogenase expression and activity varied between species within the class Clostridia. *Anaerostipes hadrus* encoded two [FeFe]-hydrogenases yet transcribed the group B genes at significantly higher levels (average 318 TPM) than its group A2 genes (33 TPM) **(Fig. 3a; Supplementary Dataset 4).** Similarly, *Gemmiger formicilis* also exhibited higher transcription of the genes encoding the group B hydrogenase (254 TPM) compared to its group A1 (139 TPM) and A2 (39 TPM) hydrogenases **(Fig. 3a; Supplementary Dataset 4)**. These findings indicate that the group B [FeFe]-hydrogenase serves as the primary fermentative hydrogenase in both species. In contrast, the opportunistic pathogens *Clostridium perfringens* and *Clostridium baratii* transcribed their prototypical fermentative group A1 [FeFe]-hydrogenase genes at much higher levels (*C. perfringens*: 305 TPM, *C. baratii*: 850 TPM) than their group B [FeFe]-hydrogenase genes (*C. perfringens:* 3.03 TPM, *C. baratii*: 0.42 TPM) **(Fig. 3a; Supplementary Dataset 4)**. These extremely fast-growing bacteria also both produced much higher levels of H_2 than the other isolates (up to 26.7% H_2) **(Fig. 3a; Fig. S1)**. These findings are consistent with biochemical and genetic studies suggesting the group A1 enzyme predominates H_2 production in *C. perfringens*[1,2]. Such *Clostridium* species appear to have evolved these exceptionally rapid group A1 [FeFe]-hydrogenases to enable vigorous growth in high nutrient conditions, such as experienced in this experimental setup. However, the metagenomic and metatranscriptomic analyses suggests they are in low abundance in most stool and biopsy samples **(Fig. 1)**. It remains unclear under which conditions such species express group B [FeFe]-hydrogenase genes. These observations suggest that species within the same phyla may employ distinct hydrogenases for similar purposes. *Dorea longicatena* generated substantial amounts of H_2, but transcriptomes yielded minimal reads mapping to metabolic genes and hence it is unclear whether its group B [FeFe]-hydrogenase is responsible **(Fig. 3a)**. In a further exception, the actinobacterium *Olsenella umbonata* did not produce detectable H_2 despite encoding and transcribing a group B [FeFe]-hydrogenase gene **(Fig. 3a)**. It is possible that its hydrogenase is active under specific conditions or alternatively this microbe internally recycles H_2 using its group A2 [FeFe]-hydrogenase.

Supplementary Note 2. AlphaFold modelling of *Bacteroides* [FeFe]-hydrogenases. AlphaFold2 suggests group B [FeFe]-hydrogenases are structurally distinct *bona fide* hydrogenases. They feature the three highly conserved sequence motifs of [FeFe]-hydrogenases, which line the binding pocket of the H-cluster (L1: T_{294}SCCPSY_{300}, L2: G_{342}PCVAKRKE_{350}, L3: E_{448}VMACEGGCISGP_{460}) **(Fig. 4b; Fig. S5)**; notably, Cys456 bridges the [4Fe4S] and catalytic di-iron site of the H-cluster, and Met450 interacts with the amine bridgehead group of the di-iron site dithiolate ligand. Structural comparison

with the well-characterized group A1 [FeFe]-hydrogenase (*C. pasteurianum* hydrogenase, CpI; PDB: 6GM2[3]) revealed that, while the H-cluster domain is largely conserved, the group B [FeFe]-hydrogenase is otherwise structurally unique (**Fig. 4a**). They particularly differ in their electron-relaying iron-sulfur clusters: whereas the group A enzyme contains a [2Fe2S] ferredoxin-like domain and a 3-Cys, 1-His ligated [4Fe4S] cluster[4], the group B enzyme is instead predicted to have a single ferredoxin-like domain containing 2×[4Fe4S] clusters. This ferredoxin-like domain is unusual in that its iron-sulfur clusters are distant from the main body of the enzyme as they are separated from the nearest [4Fe4S] cluster in the H-cluster domain by an edge-to-edge distance of at least 22 Å **(Fig. 4a)**. Even after accounting for conformational flexibility of the interdomain loops using AF-Cluster[5], the greater than 14 Å distance between these two clusters would likely limit the rate of electron transfer[6]. We hypothesise that the group B [FeFe]-hydrogenase interacts with soluble ferredoxins or electron donating enzymes to enable productive electron transfer. Structural predictions also indicated that the group A3 [FeFe]-hydrogenases of *Bacteroides* are a homodimer of heterotrimers that confurcate electrons from reduced ferredoxin and NADH to H_2 **(Fig. S6)**.

Supplementary Note 3. Activities and EPR characteristics of the *Bacteroides* [FeFe]-hydrogenases.

To test whether the *Bacteroides* hydrogenases were catalytically active, we expressed their group B and A3 [FeFe]-hydrogenase catalytic subunit genes in *E. coli* BL21(DE3) cells **(Table S5; Fig. S7)**, activated lysates with the H-cluster mimic $[2Fe]^{adt}$ as previously described[7-9], and tested their ability to produce H_2 using the standard methyl viologen as redox mediator and sodium dithionite as sacrificial electron donor. Their relative activity varied compared to the positive control (the fast-acting group A1 [FeFe]-hydrogenase of *Chlamydomonas reinhardtii*[9-13]; at 30% (*B. fragilis*), 8% (*B. thetaiotaomicron*), and 2% (*B. vulgatus*) **(Fig. 4c; Table S2)**; these contrasting activities likely reflect differences in the expression, maturation, or solubility of these enzymes in the heterologous host, though are unlikely to be physiologically relevant given the species these enzymes were derived from each produced comparable amounts of H_2 **(Fig. 3)**.

To measure EPR spectra, the *B. fragilis* group B [FeFe]-hydrogenase was incubated with $[2Fe]^{pdt}$, a catalytically inactive cofactor mimic known to stabilise the di-iron site in an EPR-active mixed valent $Fe^{I}Fe^{II}$ oxidation. This reflects the $[2Fe]^{pdt}$ cofactor mimic lacks the nitrogen bridgehead of $[2Fe]^{adt}$, hampering catalysis and promoting the build-up of the EPR-active H-cluster resting state, H_{ox}. Cell suspensions displayed a partially resolved rhombic EPR signal with g_1 = 2.101 and g_2 2.053 (**Fig. 4d**). The observed *g*-values are in good agreement with previously reported [FeFe]-hydrogenases, typically with values above g = 2 (see **Table S6**). This suggests formation of the H-cluster in an H_{ox}-like state and support the notion that the H-cluster of the group B [FeFe]-hydrogenase has an electronic structure similar to the distantly related prototypical group A enzymes **(Table S6)**. The third *g*-value (g_3) is expected to be positioned at around 2.010-2.009, but it cannot be distinctly resolved due to strong overlap with BL21(DE3) cell background signals. Whole-cell samples of the *B.*

thetaiotaomicron group B [FeFe]-hydrogenase, the second most active gut hydrogenase in the activity screening, did not exhibit clear H-cluster signals when incubated with $[2Fe]^{pdt}$ and exhibited a spectrum equivalent to that of BL21(DE3) cells not expressing any [FeFe]-hydrogenase (data not shown). The absence of any discernible EPR signal attributable to the H-cluster in these samples is potentially due to the formation of H-cluster states that are EPR-silent, but is more likely due to the low solubility and thereby low concentration of holo-enzyme in the whole-cell mixture.

Supplementary Note References

1 Kaji, M. *et al.* The hydA gene encoding the H2-evolving hydrogenase of Clostridium perfringens: molecular characterization and expression of the gene. *FEMS Microbiology Letters* **181**, 329-336 (1999). https://doi.org/10.1111/j.1574-6968.1999.tb08863.x

2 Morra, S., Mongili, B., Maurelli, S., Gilardi, G. & Valetti, F. Isolation and characterization of a new [FeFe]-hydrogenase from Clostridium perfringens. *Biotechnology and Applied Biochemistry* **63**, 305-311 (2016).

3 Duan, J. *et al.* Crystallographic and spectroscopic assignment of the proton transfer pathway in [FeFe]-hydrogenases. *Nature Communications* **9**, 4726 (2018).

4 Mulder, D. W. *et al.* Insights into [FeFe]-hydrogenase structure, mechanism, and maturation. *Structure* **19**, 1038-1052 (2011).

5 Wayment-Steele, H. K. *et al.* Predicting multiple conformations via sequence clustering and AlphaFold2. *Nature* **625**, 832-839 (2024).

6 Page, C. C., Moser, C. C., Chen, X. & Dutton, P. L. Natural engineering principles of electron tunnelling in biological oxidation–reduction. *Nature* **402**, 47-52 (1999).

7 Berggren, G. *et al.* Biomimetic assembly and activation of [FeFe]-hydrogenases. *Nature* **499**, 66-69 (2013).

8 Greening, C. *et al.* Minimal and hybrid hydrogenases are active from archaea. *Cell* **187**, 3357-3372. e3319 (2024).

9 Land, H. *et al.* Discovery of novel [FeFe]-hydrogenases for biocatalytic H 2-production. *Chemical Science* **10**, 9941-9948 (2019).

10 Khanna, N., Esmieu, C., Mészáros, L. S., Lindblad, P. & Berggren, G. In vivo activation of an [FeFe] hydrogenase using synthetic cofactors. *Energy & Environmental Science* **10**, 1563-1567 (2017).

11 Le Cloirec, A. *et al.* A di-iron dithiolate possessing structural elements of the carbonyl/cyanide sub-site of the H-centre of Fe-only hydrogenase. *Chemical Communications*, 2285-2286 (1999).

12 Lyon, E. J., Georgakaki, I. P., Reibenspies, J. H. & Darensbourg, M. Y. Carbon monoxide and cyanide ligands in a classical organometallic complex model for Fe-only hydrogenase. *Angewandte Chemie International Edition* **38**, 3178-3180 (1999).

13 Schmidt, M., Contakes, S. M. & Rauchfuss, T. B. First generation analogues of the binuclear site in the Fe-only hydrogenases: Fe2 (μ-SR) 2 (CO) 4 (CN) 2 2. *J. Am. Chem. Soc* **121**, 9736-9737 (1999).

Supplementary Datasets

Supplementary Dataset 1. "*Supplementary Dataset 1 - Hydrogenase abundance and expression in gut metagenomes and metatranscriptomes.xlsx*". A comprehensive analysis of the abundance, transcription and taxonomic origin of genes encoding hydrogenases and H_2-cycling-related metabolic genes throughout stool metagenome and metatranscriptome samples and biopsy metagenome samples (with accompanying ENA run accession numbers). The data is organised as per the following tabs:

1. "**Biopsy Sample Metadata"**
   - The gender, region of biopsy sample and accession numbers for all *biopsy* samples used in this study.
2. **"Stool MetaG Hyd-sbg cpg"**
   - Abundance values, expressed as copies per genome (cpg), of genes encoding hydrogenase subgroups throughout the *stool metagenome* samples.
3. **"Stool MetaG Hyd-sbg RPKM"**
   - Abundance values, expressed as reads per kilobase million (RPKM), of genes encoding hydrogenase subgroups throughout the *stool metagenome* samples.
4. **"Stool MetaG Hyd-mg Analysis"**
   - Presence/absence analysis and average abundance values (cpg and RPKM) of groupings of the genes encoding the main hydrogenase groups ([NiFe]- and [FeFe]-hydrogenase) throughout the *stool metagenome* samples.
5. **"Biopsy MetaG Hyd-sbg cpg"**
   - Abundance values, expressed as copies per genome (cpg), of genes encoding hydrogenase subgroups throughout the *biopsy metagenome* samples.
6. **"Biopsy MetaG Hyd-sbg RPKM"**
   - Abundance values, expressed as reads per kilobase million (RPKM), of genes encoding hydrogenase subgroups throughout the *biopsy metagenome* samples.
7. **"Biopsy MetaG Hyd-mg Analysis"**
   - Presence/absence analysis and average abundance values (cpg and RPKM) of groupings of the genes encoding the main hydrogenase groups ([NiFe]- and [FeFe]-hydrogenase) throughout the *biopsy metagenome* samples.
8. **"Stool MetaT Hyd-sbg RPKM"**
   - Transcription values, expressed as reads per kilobase million (RPKM), of genes encoding hydrogenase subgroups throughout the *stool metatranscriptome* samples. $Type\ equation\ here.$
9. **"Stool MetaT Hyd-mg Analysis"**
   - Transcribed/Not transcribed analysis and average transcription values (RPKM) of genes encoding the main hydrogenase groupings ([NiFe]- and [FeFe]-hydrogenase) throughout the *stool metatranscriptome* samples.

10. **“Stool MetaT FuncGenes Analysis”**
    - Transcribed/not transcribed analysis and transcription values (RPKM), of H_2-cycling-related metabolic genes throughout the *stool metatranscriptome* samples.
11. **“Stool & Biopsy MetaG FuncGenes”**
    - Presence/absence analysis and abundance values (cpg & RPKM), of H_2-cycling-related metabolic genes throughout the *stool* (yellow) & *biopsy* (orange) *metagenome* samples.
12. **“Stool v Biopsy MetaG Av. cpg”**
    - Comparative analysis of the average abundance (cpg) of genes encoding hydrogenase subgroups between *stool* vs *biopsy* metagenomes
13. **“Transcr-Abun FuncGenes RPKM”**
    - Transcription and abundance values (RPKM), and corresponding ratios, of H_2-cycling-related metabolic genes across paired *stool metagenome* and *metatranscriptome* samples.
14. **“Transcr-Abun Hyd-sbg RPKM”**
    - Transcription and abundance values (RPKM), and corresponding ratios, of genes encoding hydrogenase subgroups across paired *stool metagenome* and *metatranscriptome* samples.
15. **“Transcr-Abun Hyd-mg RPKM”**
    - Transcription and abundance values (RPKM), and corresponding ratios, of groupings of genes encoding the main hydrogenase subgroups across paired *stool metagenome* and *metatranscriptome* samples.
16. **“Genus Hyd-sbg sum(RPKM)”**
    - The genera with the highest abundance from *stool* and *biopsy* metagenomes, and transcriptional activity from *stool metatranscriptomes* (expressed as sum(RPKM)), of genes encoding hydrogenase subgroups as inferred from read-mapping.
17. **“Phylum abun. MetaG sum(RPKM)”**
    - Phylum-level abundance (sum(RPKM) of genes encoding hydrogenase subgroups throughout the *stool* and *biopsy metagenome* samples.

Supplementary Dataset 2. “*Supplementary Dataset 2 - Taxonomy and H_2 cycling related metabolic capacity of AusMICC gut isolates.xlsx*”. Taxonomic classifications for 812 human gut isolate genomes from the Australian Microbiome Culture Collection (AusMicc) (with accompanying ENA run accession numbers), along with a summary of the presence or absence of genes encoding hydrogenase subgroups and other H_2 cycling-related metabolic genes across these genomes. The data is organised in the following tabs:

1. **“Tax & Funcgene Hit Summary”**

- A summary of the phyla and genus level taxonomic identification of each isolate genome, along with hits to hydrogenase subgroup and other H_2 cycling related metabolic genes, along with the database information for each hit.

2. **"Gene Count and % Analysis"**
   - Count and percentage presence of the genes encoding hydrogenase subgroups and other H_2 cycling related metabolic genes across all isolate genomes.
3. **"Genus Analysis"**
   - The dominant genera (identified by GTDB-Tk) that encode hydrogenase subgroup and other H_2 cycling related metabolic genes across the isolate genome collection.
4. **"Phylum Analysis"**
   - The dominant phyla (identified by GTDB-Tk) that encode hydrogenase subgroup and other H_2 cycling related metabolic genes across the isolate genome collection.
5. **"GTDB-Tk IDs"**
   - GTDB-Tk-based taxonomic classification of each isolate genome.
6. **"CheckM"**
   - Quality assessment metrics obtained from CheckM for each isolate genome.
7. **"Bacteroides Hydrogenases"**
   - Number of overall hydrogenase gene hits detected to be encoded by each *Bacteroides* isolate genome.

Supplementary Dataset 3. **"***Supplementary Dataset 3 - Taxonomic classification of stool and biopsy metagenomes.xlsx*". The relative abundance (%) of microbial taxa in the stool and biopsy metagenome samples, as profiled by MetaPhlan4. Analysis of the proportion of metagenome samples that detect *Bacteroides*, and average relative abundance of *Bacteroides* within those samples is also included. The data is organised via the following tabs:

1. **"Biopsy metagenome taxonomy".**
   - The relative abundance (%) of microbial taxa detected within the *biopsy metagenome* samples. Rows represent the taxonomic clades from kingdom to species level, and columns represent individual metagenome samples. The relative abundance of *Bacteroides* across samples is highlighted in red, and analysis of abundance and prevalence of *Bacteroides* is included.
2. **"Biopsy MetaG ENA Accessions"**
   - A summary of the *biopsy metagenome* sample ID's used in the analysis, the corresponding filenames and their ENA run accession numbers that can be used to access the files.
3. **"Stool metagenome taxonomy".**
   - The relative abundance (%) of microbial taxa detected within the *stool metagenome* samples. Rows represent the taxonomic clades from kingdom to

species level, and columns represent individual metagenome samples. The relative abundance of *Bacteroides* across samples is highlighted in red, and analysis of abundance and prevalence of *Bacteroides* is included.

Supplementary Dataset 4. "*Supplementary Dataset 4 - AusMiCC Gut Isolate transcriptome summaries.xlsx*". Summaries of the transcriptomic analysis of all 18 gut isolates obtained from AusMiCC, including transcripts per million values for all transcribed and annotated genes, and accompanying KEGG ID's and modules. The data is organised into the following tabs:

1. **"Isolate Genome IDs & Accessions"**
   - A list of the AusMiCC isolate IDs and the corresponding species identification, ENA run accession numbers for the isolate genomes, and ENA run accession numbers for the transcriptome (RNA-seq file) of each isolate replicate (n=3).
2. **"B. caccae"**
   - A list of all transcribed and annotated genes and their quantification (transcripts per million, TPM) from triplicate cultures of *Bacteroides caccae*, including KEGG ID and module information, and the DIAMOND homology-based identification of the hydrogenase and related H_2 cycling genes.
3. **"B. dorei"**
   - A list of all transcribed and annotated genes and their quantification (transcripts per million, TPM) from triplicate cultures of *Bacteroides dorei*, including KEGG ID and module information, and the DIAMOND homology-based identification of the hydrogenase and related H_2 cycling genes.
4. **"B. faecis"**
   - A list of all transcribed and annotated genes and their quantification (transcripts per million, TPM) from triplicate cultures of *Bacteroides faecis*, including KEGG ID and module information, and the DIAMOND homology-based identification of the hydrogenase and related H_2 cycling genes.
5. **"B. fragilis"**
   - A list of all transcribed and annotated genes and their quantification (transcripts per million, TPM) from triplicate cultures of *Bacteroides fragilis*, including KEGG ID and module information, and the DIAMOND homology-based identification of the hydrogenase and related H_2 cycling genes.
6. **"B. plebius"**
   - A list of all transcribed and annotated genes and their quantification (transcripts per million, TPM) from triplicate cultures of *Bacteroides plebius*, including KEGG ID and module information, and the DIAMOND homology-based identification of the hydrogenase and related H_2 cycling genes.

7. **"B. theta"**
   - A list of all transcribed and annotated genes and their quantification (transcripts per million, TPM) from triplicate cultures of *Bacteroides thetaiotaomicron*, including KEGG ID and module information, and the DIAMOND homology-based identification of the hydrogenase and related H_2 cycling genes.
8. **"B. vulgatus"**
   - A list of all transcribed and annotated genes and their quantification (transcripts per million, TPM) from triplicate cultures of *Bacteroides vulgatus*, including KEGG ID and module information, and the DIAMOND homology-based identification of the hydrogenase and related H_2 cycling genes.
9. **"C. baratii"**
   - A list of all transcribed and annotated genes and their quantification (transcripts per million, TPM) from triplicate cultures of *Clostridium baratii*, including KEGG ID and module information, and the DIAMOND homology-based identification of the hydrogenase and related H_2 cycling genes.
10. **"C. perfringens"**
    - A list of all transcribed and annotated genes and their quantification (transcripts per million, TPM) from triplicate cultures of *Clostridium perfringens*, including KEGG ID and module information, and the DIAMOND homology-based identification of the hydrogenase and related H_2 cycling genes.
11. **"A. hadrus"**
    - A list of all transcribed and annotated genes and their quantification (transcripts per million, TPM) from triplicate cultures of *Anaerostipes hadrus*, including KEGG ID and module information, and the DIAMOND homology-based identification of the hydrogenase and related H_2 cycling genes.
12. **"D. longicatena"**
    - A list of all transcribed and annotated genes and their quantification (transcripts per million, TPM) from triplicate cultures of *Dorea longicatena*, including KEGG ID and module information, and the DIAMOND homology-based identification of the hydrogenase and related H_2 cycling genes.
13. **"G. formicilis"**
    - A list of all transcribed and annotated genes and their quantification (transcripts per million, TPM) from triplicate cultures of *Gemmiger formicilis*, including KEGG ID and module information, and the DIAMOND homology-based identification of the hydrogenase and related H_2 cycling genes.
14. **"N. rosorum"**
    - A list of all transcribed and annotated genes and their quantification (transcripts per million, TPM) from triplicate cultures of *Necropsobacter rosorum*, including KEGG

ID and module information, and the DIAMOND homology-based identification of the hydrogenase and related H_2 cycling genes.

15. **"C. aerofaciens"**
    - A list of all transcribed and annotated genes and their quantification (transcripts per million, TPM) from triplicate cultures of *Collinsella aerofaciens*, including KEGG ID and module information, and the DIAMOND homology-based identification of the hydrogenase and related H_2 cycling genes.
16. **"O. umbonata"**
    - A list of all transcribed and annotated genes and their quantification (transcripts per million, TPM) from triplicate cultures of *Olsenella umbonata*, including KEGG ID and module information, and the DIAMOND homology-based identification of the hydrogenase and related H_2 cycling genes.
17. **"F. varium"**
    - A list of all transcribed and annotated genes and their quantification (transcripts per million, TPM) from triplicate cultures of *Fusobacterium varium*, including KEGG ID and module information, and the DIAMOND homology-based identification of the hydrogenase and related H_2 cycling genes.
18. **"C. mitsuokai"**
    - A list of all transcribed and annotated genes and their quantification (transcripts per million, TPM) from triplicate cultures of *Catenibacterium mitsuokai*, including KEGG ID and module information, and the DIAMOND homology-based identification of the hydrogenase and related H_2 cycling genes.
19. **"B. longum"**
    - A list of all transcribed and annotated genes and their quantification (transcripts per million, TPM) from triplicate cultures of *Bifidobacterium longum*, including KEGG ID and module information, and the DIAMOND homology-based identification of the hydrogenase and related H_2 cycling genes.
20. **"TPM Summary - All"**
    - The combined raw transcriptomic information expressed as TPM (outputted by Salmon) for each isolate.
21. **"Bacteroides Hyd TPM Summary"**
    - A quantified transcription summary of the hydrogenase genes encoded by *Bacteroides* isolates.
22. **"Av. Phyla TPM - DIAMOND genes"**
    - Average transcript abundance (TPM) of hydrogenase subgroup and other H_2 cycling related metabolic genes across all encoding isolates within each phylum.
23. **"Isolate genome DIAMOND hits"**

- A list of all hits to each isolate genome identified through homology-based searches using DIAMOND against an in-house database containing hydrogenase and other H_2 cycling related metabolic protein sequences.

24. **"Bacteroides KEGG ID"**
    - Transcript abundance data (TPM) for the KEGG-annotated genes across Bacteroides isolate genomes.

Supplementary Dataset 5. "*Supplementary Dataset 5 – Fermentation mass balance*". A summary of substrate consumption, product formation, and growth parameters of *Bacteroides fragilis* grown on minimal medium (mBMM) in the presence and absence of hemin. The data is organised in the following tabs:

1. **"H2 & Glucose Concs Heme"**
    - H_2 production (ppm), glucose consumption (mg/100 mL), and growth (OD_{600}) of *B. fragilis* with hemin.
2. **"H2 & Glucose Concs No Heme"**
    - H_2 production (ppm), glucose consumption (mg/100 mL), and growth (OD_{600}) of *B. fragilis* without hemin.
3. **"SCFAs"**
    - Short-chain fatty acids (μM) production by *B. fragilis* with and without hemin.
4. **"Fermentation balance"**
    - Proportion of carbon and electrons converted into each fermentation product by *B. fragilis*. This was only calculated in the presence of hemin given it was possible to account for all reducing equivalents (99.5 to 114%) in the fermentation products in this condition, but not in the absence of hemin where other unmeasured metabolites (e.g. fumarate) were likely formed.

Supplementary Dataset 6. "*Supplementary Dataset 6 - Hydrogenase gene abundance in stool metagenomes of healthy vs unhealthy individuals*". A summary of the relative abundances (in copies per genome, cpg) of hydrogenase subgroup genes and other H_2-cycling related metabolic genes across stool metagenomes of healthy individuals compared to those experiencing various chronic disease states. The data is organised in the following tabs:

5. **"FuncGenes abund."**
    - Relative abundances (cpg) of H_2-cycling related functional/metabolic genes for each stool metagenome sample, along with their health status (healthy or various chronic disease states).
6. **"FuncGenes abund. across disease"**
    - A summary of the average relative abundance (mean cpg + standard deviation) of hydrogenase main groups and various H_2 cycling related metabolic genes for the healthy state, and for each disease state.
7. **"Hyd-sbgr abund."**

- Relative abundances (cpg) of hydrogenase subgroup genes for each stool metagenome sample, along with their health status (healthy or various chronic disease states).

8. **“Hyd-sbgr abund. across diseases”**
    - A summary of the average relative abundance (mean cpg + standard deviation) of hydrogenase subgroup genes for the healthy state, and for each disease state.

Supplementary Tables

Table S1. Taxonomy and encoded hydrogenase genes of the 19 gut isolates from Australian Microbiome Culture Collection (AusMiCC) used for growth, H_2 production, and transcriptome analyses.

Species	Isolate ID	Encoded hydrogenase genes
Clostridium perfringens	CC01445	Group B [FeFe]-hydrogenase<br>Group B [FeFe]-hydrogenase<br>Group A1 [FeFe]-hydrogenase<br>Group A1 [FeFe]-hydrogenase
Clostridium baratii	CC01452	Group B [FeFe]-hydrogenase<br>Group A1 [FeFe]-hydrogenase
Fusobacterium varium	CC01421	Group A1 [FeFe]-hydrogenase<br>Group A1 [FeFe]-hydrogenase<br>Group A3 [FeFe]-hydrogenase
Necropsobacter rosorum	CC01403	Group 1c [NiFe]-hydrogenase<br>Group 4a [NiFe]-hydrogenase
Bacteroides caccae	CC01389	Group B [FeFe]-hydrogenase<br>Group A3 [FeFe]-hydrogenase
Bacteroides thetaiotaomicron	CC00765	Group B [FeFe]-hydrogenase<br>Group A3 [FeFe]-hydrogenase
Bacteroides dorei	CC01440	Group B [FeFe]-hydrogenase
Bacteroides faecis	CC01412	Group B [FeFe]-hydrogenase<br>Group A3 [FeFe]-hydrogenase
Bacteroides vulgatus	CC01422	Group B [FeFe]-hydrogenase
Bacteroides fragilis	CC01400	Group B [FeFe]-hydrogenase
Bacteroides plebius	CC01397	Group B [FeFe]-hydrogenase
Gemmiger formicilis	CC00311	Group B [FeFe]-hydrogenase<br>Group A1 [FeFe]-hydrogenase<br>Group A2 [FeFe]-hydrogenase
Dorea longicatena	CC00515	Group B [FeFe]-hydrogenase<br>Group A2 [FeFe]-hydrogenase
Anaerostipus hadrus	CC00501	Group B [FeFe]-hydrogenase<br>Group B [FeFe]-hydrogenase<br>Group A2 [FeFe]-hydrogenase
Olsenella umbonata	CC00540	Group B [FeFe]-hydrogenase<br>Group A2 [FeFe]-hydrogenase
Collinsella aerofaciens	CC00529	Group 4a [NiFe]-hydrogenase<br>Group 4e [NiFe]-hydrogenase<br>Group A2 [FeFe]-hydrogenase
Bifidobacterium longum	CC00565	None
Catenibacterium mitsuokai	CC00599	None
Bacteroides stercoris	CC00654	None

Table S2. Features and activities of the [FeFe]-hydrogenases heterologously expressed and semisynthetically matured in *E. coli*.

Group	Subclass	Species	[FeS] Architecture	Size (kDa)	% Activity*
B	M3a	*Bacteroides fragilis* (Bf)	3 x [4Fe-4S], 1 x [2Fe-2S]	55	34 ± 6
B	M3a	*Bacteroides vulgatus* (Bv)	3 x [4Fe-4S], 1 x [2Fe-2S]	65	1.9 ± 0.3
B	M3a	*Bacteroides thetaiotaomicron* (Bt)	3 x [4Fe-4S], 1 x [2Fe-2S]	55	7.6 ± 1.3
A3	M3	*Bacteroides thetaiotaomicron* (Bt)	3 x [4Fe-4S], 1 x [2Fe-2S]	65	0.22 ± 0.04

* with respect to the H_2 evolution rates of the *Chlamydomonas reinhardtii* group A1 [FeFe]-hydrogenase (*Cr*HydA1).

Table S3. Previously reported *g*-values for [FeFe]-hydrogenases matured with $[2Fe]^{pdt}$, i.e. H_{ox}-$[2Fe]^{pdt}$ state.

Group	Subclass	Species	g_1	g_2	g_3
A1	M1	*Chlamydomonas reinhardtii* (*Cr*HydA1)[14]	2.094	2.039	1.998
A1	M3	*Clostridium pasteurianum* (*Cp*I)[15]	2.092	2.039	2.000
A1	M2	*Solobacterium moorei* (*Sm*HydA)[4]	2.100	2.040	2.010
A1	M2	*Desulfovibrio desulfuricans* (*Dd*H)[16]	2.095	2.041	1.998
**B**	**M3a**	***Bacteroides fragilis* (*Bf*HydM, this study)**	**2.101**	**2.053**	**Not detected**
C	M2f	*Thermotoga maritima* (*Tm*HydS)[17]	2.108	2.043	2.000
D	M2e	*Thermoanaerobacter mathranii* (*Tam*HydS)[4]	2.106	2.051	2.010

Supplementary figures

Figure S1. Growth (OD_{600}, green line) and H_2 production (ppm, pink line, n = 3) of 18 human gut isolates obtained from AusMiCC over time. The phylum of each isolate is represented by the coloured bar below each graph. The dashed red line represents the lower detection limit of the gas chromatograph.

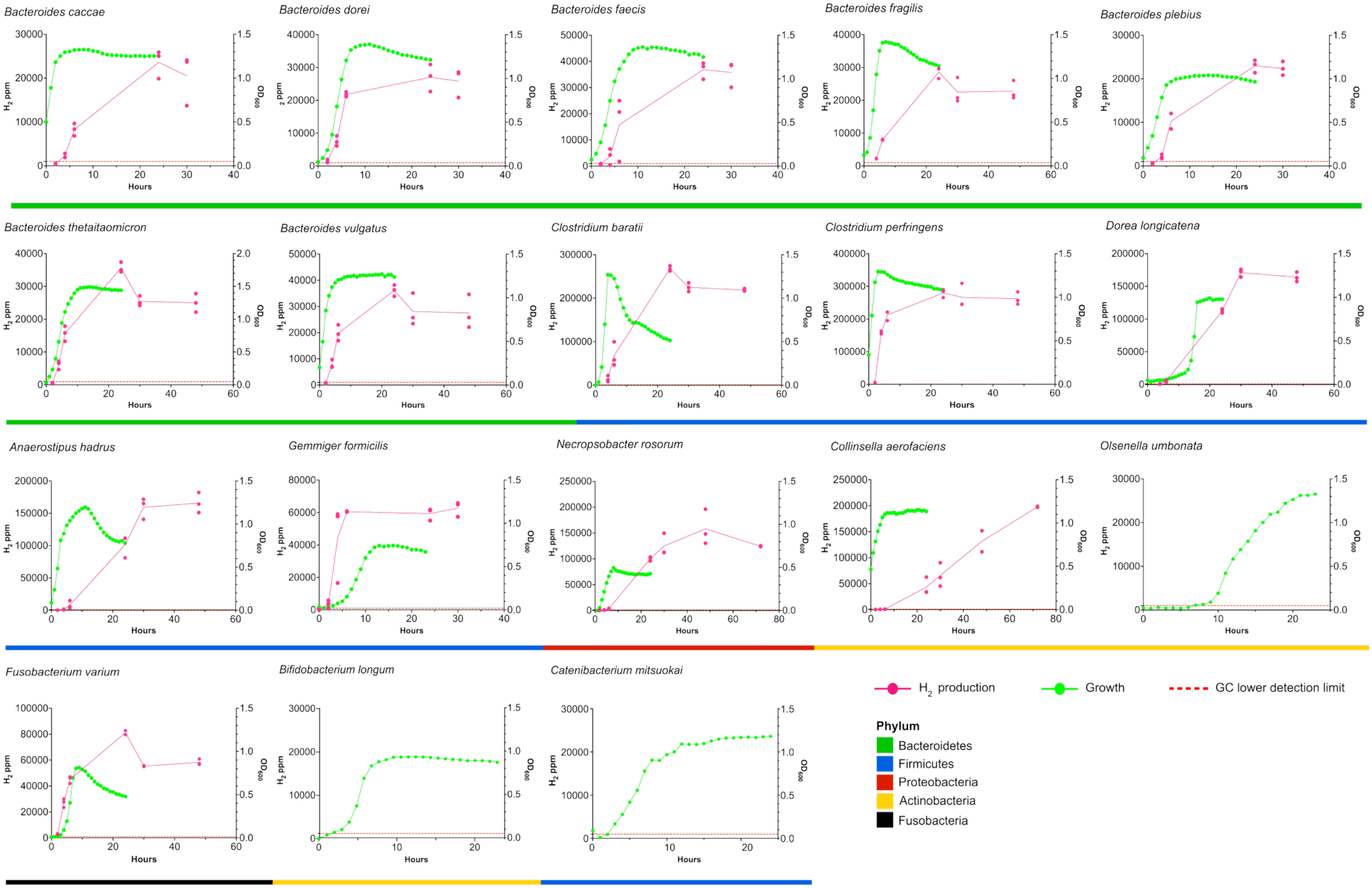
Bacteroides caccae
Bacteroides dorei
Bacteroides faecis
Bacteroides fragilis
Bacteroides plebius
Bacteroides thetaitaomicron
Bacteroides vulgatus
Clostridium baratii
Clostridium perfringens
Dorea longicatena
Anaerostipus hadrus
Gemmiger formicilis
Necropsobacter rosorum
Collinsella aerofaciens
Olsenella umbonata
Fusobacterium varium
Bifidobacterium longum
Catenibacterium mitsuokai
H_2 ppm
OD_{600}
Hours
H_2 production
Growth
GC lower detection limit
Phylum
Bacteroidetes
Firmicutes
Proteobacteria
Actinobacteria
Fusobacteria

Figure S2. H_2 production (ppm) over time measured for eight *Bacteroides* isolates (n = 3) that encode or lack detectable group B [FeFe]-hydrogenases genes. The *B. stercoris* isolate does not encode group B [FeFe]-hydrogenase genes, in contrast to all other *Bacteroides* isolates that encode and transcribe them, based on previously obtained data **(Fig 3, Table S4, S5 & S9).**

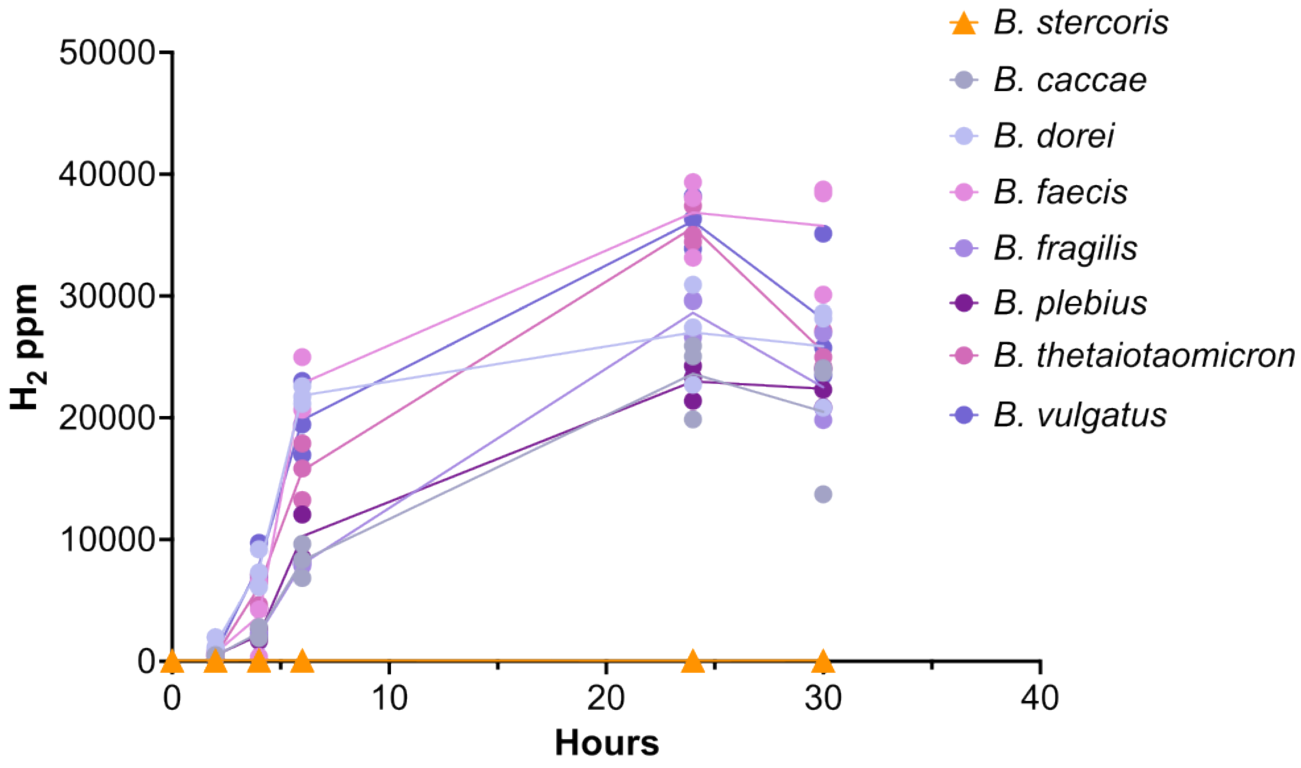

Figure S3. AlphaFold2 predicted protein structures. AlphaFold2 confidence scores for the two protein models made in this study. The top ranked model is shown and coloured according to their predicted local distance difference test (pLDDT) score, and their corresponding predicted aligned error (PAE) plots shown below. Portions of the *Bt*HydABC PAE plot axis are labelled according to their subunit identities. PAE plots were generated using PAE Viewer web server[128]. ipTM: interface predicted template modelling score. pTM: predicted template modelling score.

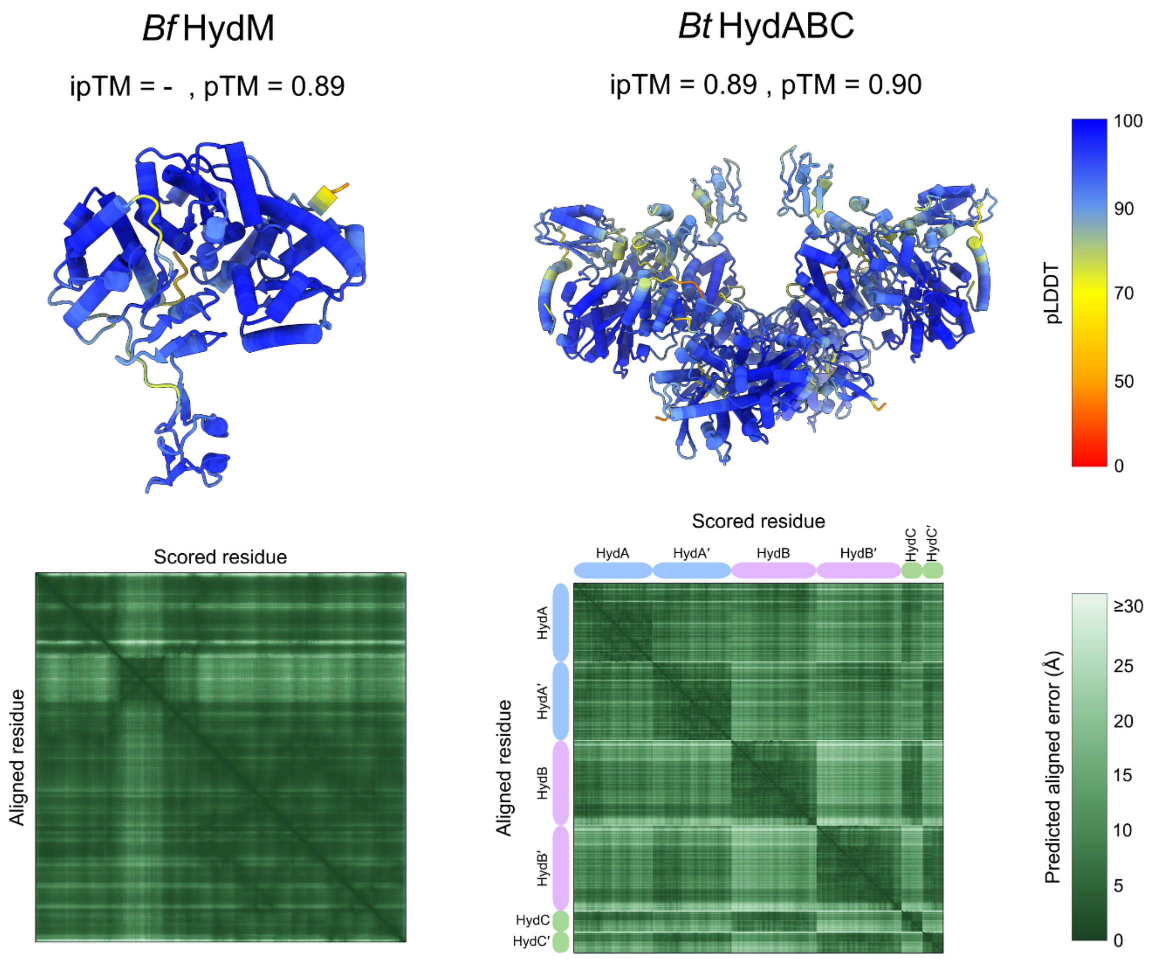

Figure S4. Process for modelling putative cofactors into the predicted structure of the *B. fragilis* group B [FeFe]-hydrogenase predicted structure. (a) Overview of strategy used for modelling cofactors into apo *Bf*HydM. Experimental structures chosen from FoldSeek output summarised on the right with their corresponding positional overlap and structural overlap (TM-score and RMSD). **(b)** Superposition of apo *Bf*HydM against overlapping portions of experimental structures chosen from FoldSeek. The PDBs were chosen to act as a template for cofactor modelling based on their (i) presence of experimentally observed cofactors and (ii) structural similarity to portions of apo *Bf*HydM, especially at conserved cysteine residues. **(c)** the putative H-cluster pocket in *Bf*HydM is highly similar to the structural architecture of that seen in the group A1 [FeFe]-hydrogenase of *Clostridium pasteurianum* (*Cp*I; PDB: 8ALN[120]). Conserved H-cluster binding residues between the two structures overlay near identically, allowing for minimal manual repositioning of the H-cluster into *Bf*HydM. **(d)** Putative iron-sulfur cluster pockets (A1 and A2) in *Bf*HydM share some structural similarity to those seen in group A1 [FeFe]-hydrogenase from *Cp*I, but with larger divergence compared to the aforementioned H-cluster. The iron-sulfur clusters required manual repositioning to coordinate with the cysteines and bond lengths were checked as being biochemically reasonable in the UCSF ChimeraX software. **(e)** Putative iron-sulfur cluster pockets (A3 and A4) in *Bf*HydM share near identical structural similarity to those seen in *Cp*I. Iron-sulfur clusters were transposed into *Bf*HydM without any manual repositioning from their relative positions observed in the *Cp*I structure.

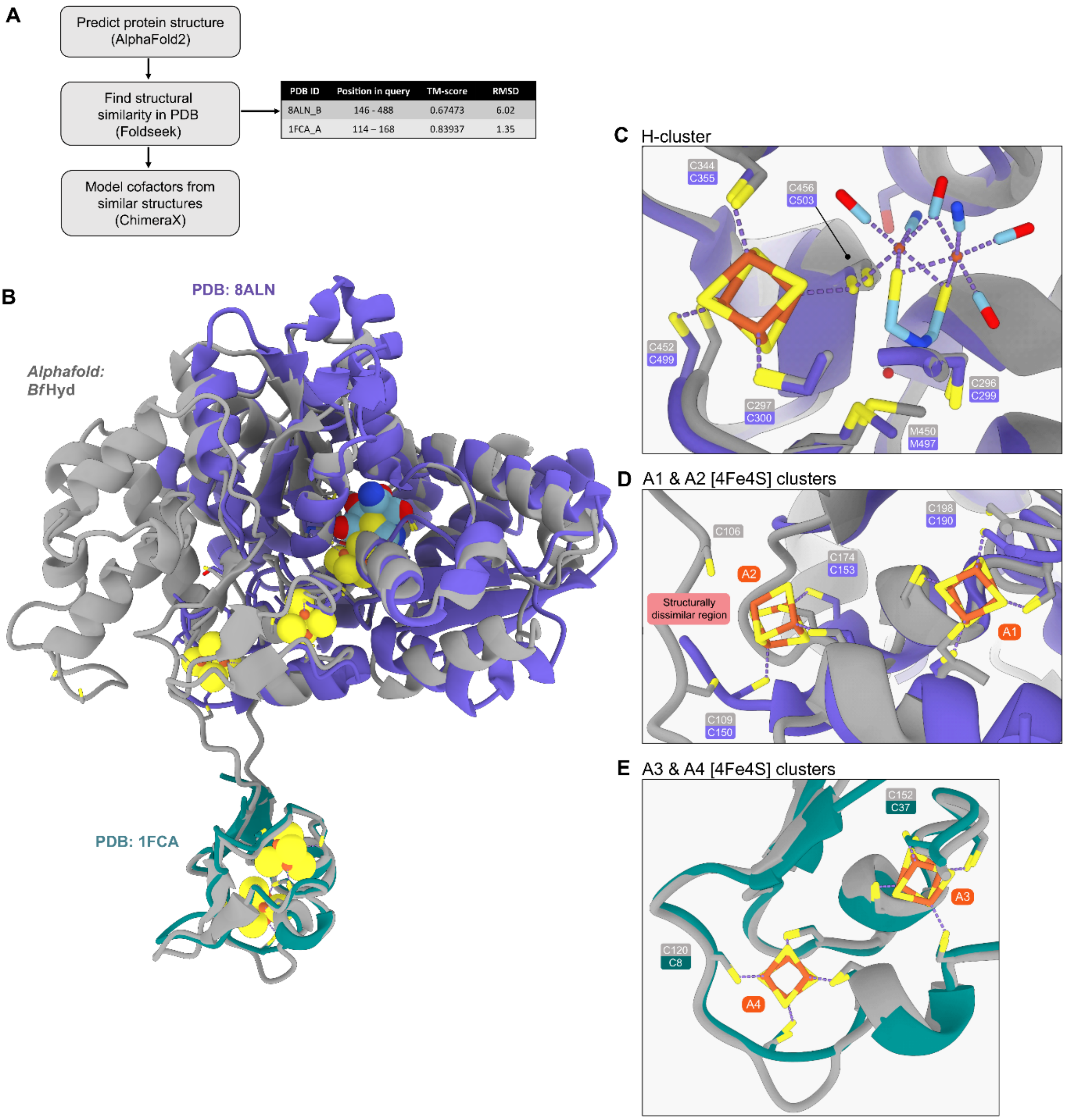
A
Predict protein structure (AlphaFold2)
Find structural similarity in PDB (Foldseek)
Model cofactors from similar structures (ChimeraX)
PDB ID | Position in query | TM-score | RMSD
8ALN_B | 146 - 488 | 0.67473 | 6.02
1FCA_A | 114 – 168 | 0.83937 | 1.35
B
PDB: 8ALN
Alphafold: BfHyd
PDB: 1FCA
C H-cluster
C344 C355
C456 C503
C452 C499
C297 C300
C296 C299
M450 M497
D A1 & A2 [4Fe4S] clusters
C106
C198 C190
C174 C153
A2
Structurally dissimilar region
A1
C109 C150
E A3 & A4 [4Fe4S] clusters
C152 C37
A3
C120 C8
A4

Figure S5. AlphaFold2 structural predictions of group B [FeFe]-hydrogenases from the *Bacteroides*. (a) Sequence conservation of Group B [FeFe]-hydrogenase homologs between *Bacteroides fragilis*, *Bacteroides thetaiotaomicron*, and *Bacteroides vulgatus*. Multiple sequence alignment was performed with ClustalO v1.2.4 and visualised with the ESPript 3.0 web server[129,130]. **(b)** Top-ranked AlphaFold2 models of the *B. thetaiotaomicron* and *B. vulgatus* group B [FeFe]-hydrogenase, coloured by pLDDT. The low pLDDT scoring N-term portion of *Bv*Hyd is possibly an intrinsically disordered domain. **(c)** Superposition of all three *Bacteroides* Group B [FeFe]-hydrogenases showing overall structural conservation, except for the N-terminal portion of *Bv*HydM which is predicted to be largely disordered.

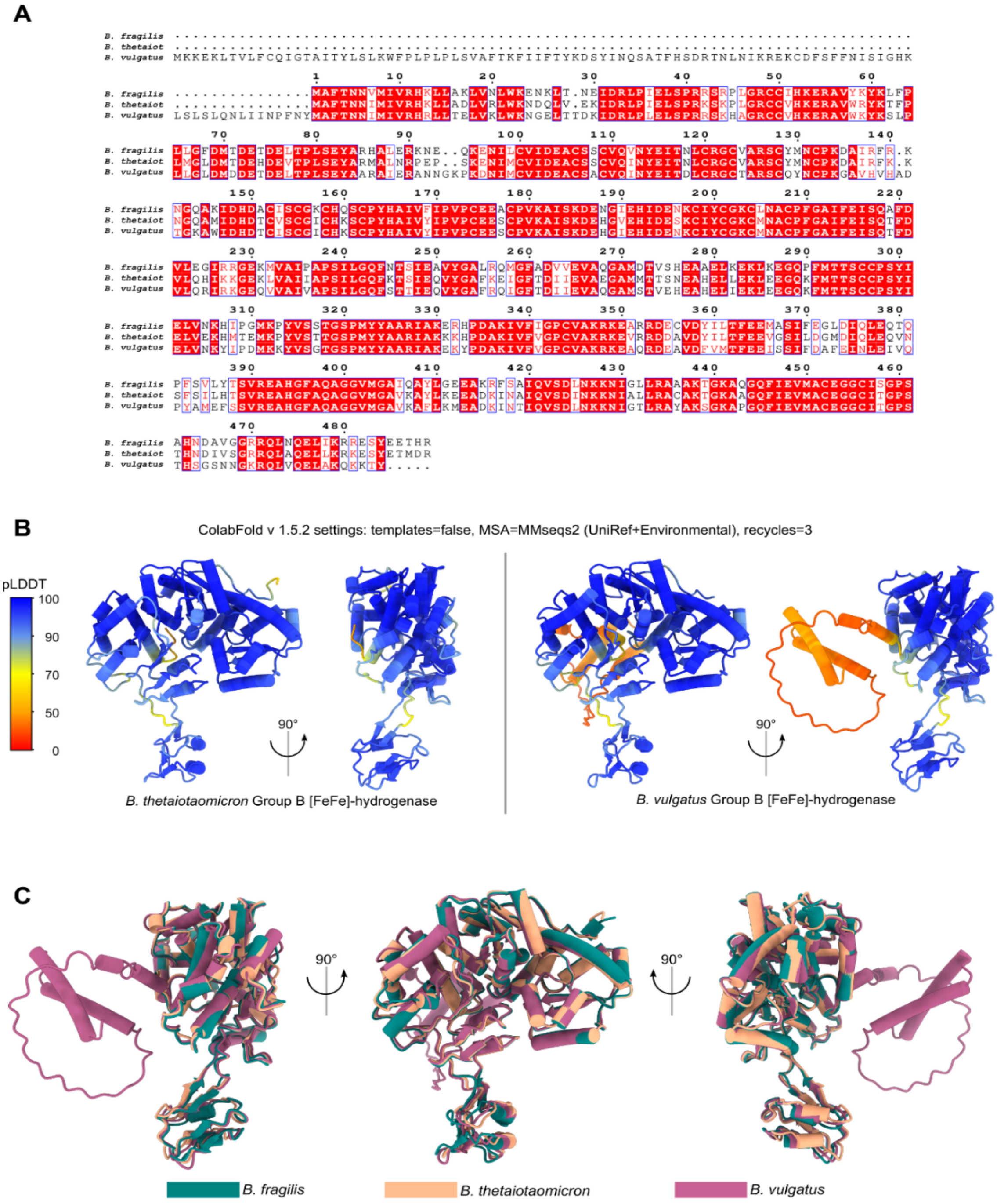

Figure S6. Predicted structure of the group A3 [FeFe]-hydrogenase complex from *Bacteroides thetaiotaomicron*. (a) Top and side view of the AlphaFold2 predicted structure. **(b)** Superposition of HydABC components with structures of *Acetobacterium woodii* HydABC (PDB ID: 8A5E[64]), *Clostridium pasteurianum* [FeFe]-hydrogenase (PDB ID: 1FEH[40]), and *Clostridium acidurici* ferredoxin (PDB ID: 1FCA[121]). The structural similarity between these proteins and *Bt*HydABC allowed for homology modelling of cofactors. **(c)** Putative cofactors and enzymatic reactions of *Bt*HydABC. Cofactors are positioned based on homology models and labelled according to their subunit identity. **(d)** Structure of the putative FMN and NADH binding site in the AlphaFold structure (left) compared to the same site in the experimental structure of *A. woodii* HydB (right). A trio of phenylalanine residues which form a π-stacking "clamp" around the adenine moiety of NADH is conserved in both structures. RMSD: root-mean-square-deviation. Å: ångström. FMN: flavin mononucleotide. NADH: nicotinamide adenine dinucleotide (reduced). NAD^+: nicotinamide adenine dinucleotide (oxidized). Fd_{red}: reduced ferredoxin. Fd_{ox}: oxidized ferredoxin.

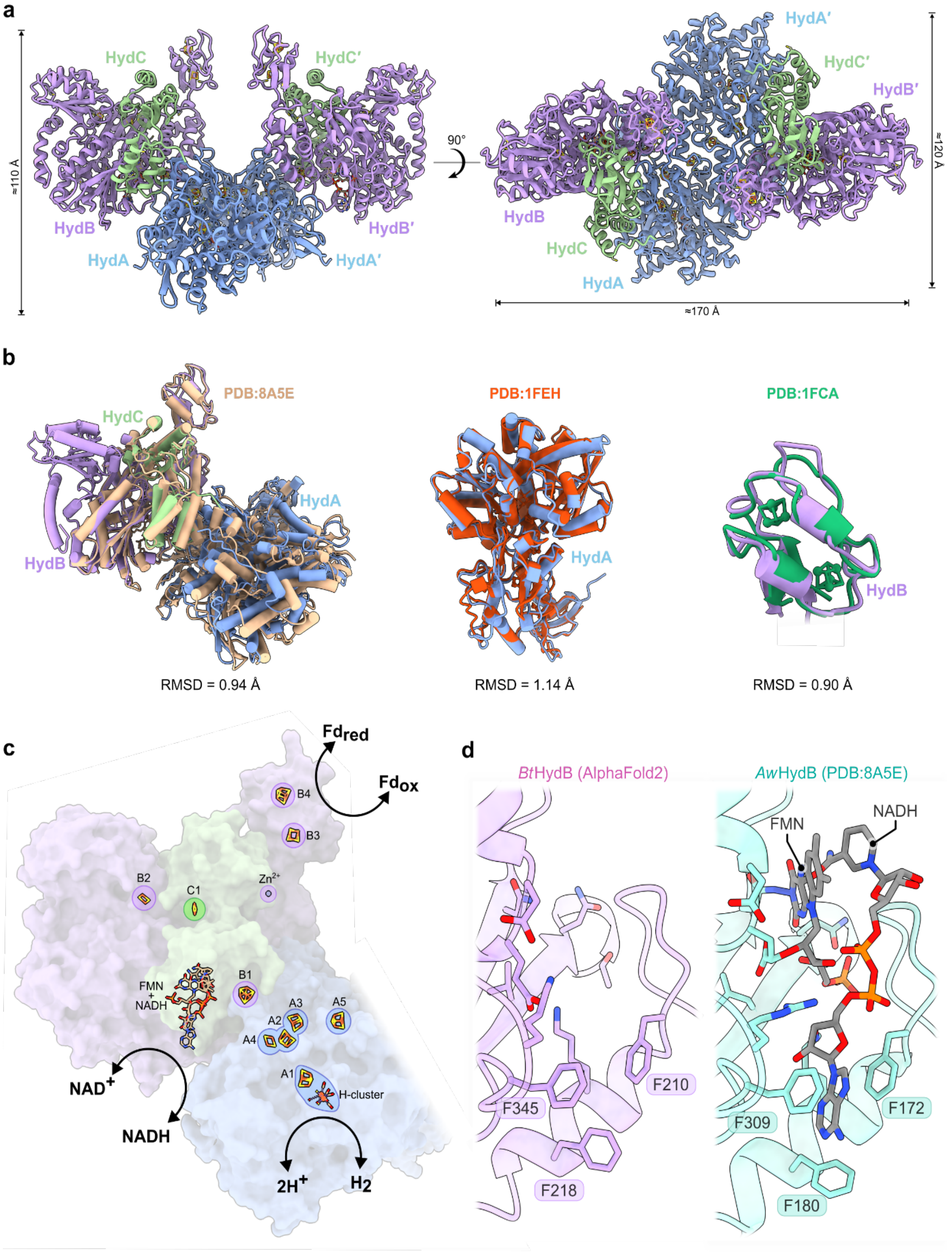

a
HydC
HydC′
HydB
HydB′
HydA
HydA′
90°
≈110 Å
≈120 Å
≈170 Å
b
PDB:8A5E
PDB:1FEH
PDB:1FCA
RMSD = 0.94 Å
RMSD = 1.14 Å
RMSD = 0.90 Å
c
Fdred
Fdox
B4
B3
B2
C1
Zn2+
FMN + NADH
B1
A3
A5
A2
A4
A1
H-cluster
NAD+
NADH
2H+
H2
d
BtHydB (AlphaFold2)
AwHydB (PDB:8A5E)
FMN
NADH
F345
F210
F218
F309
F172
F180

Figure S7. SDS-PAGE visualising the molecular weights of the heterologously expressed [FeFe]-hydrogenases from *Bacteroides*. Expression constructs with verified sequences were used to transform chemically competent *E. coli* BL21(DE3). Protein bands are shown from before induction with IPTG (B), after induction (Name-Subclass and with the expected kDa size in parenthesis), and lysate or supernatant after cell lysis and centrifugation (L). The bands in each after-induction lane corresponded well with the expected molecular weights in kDa. Three gut-associated [FeFe]-hydrogenases (**Bf-M3a**, **Bt-M3,** and **Bt-M3a**) exhibited high levels of expression and low to moderate solubility while **Bv-M3a** had poor expression and solubility levels.

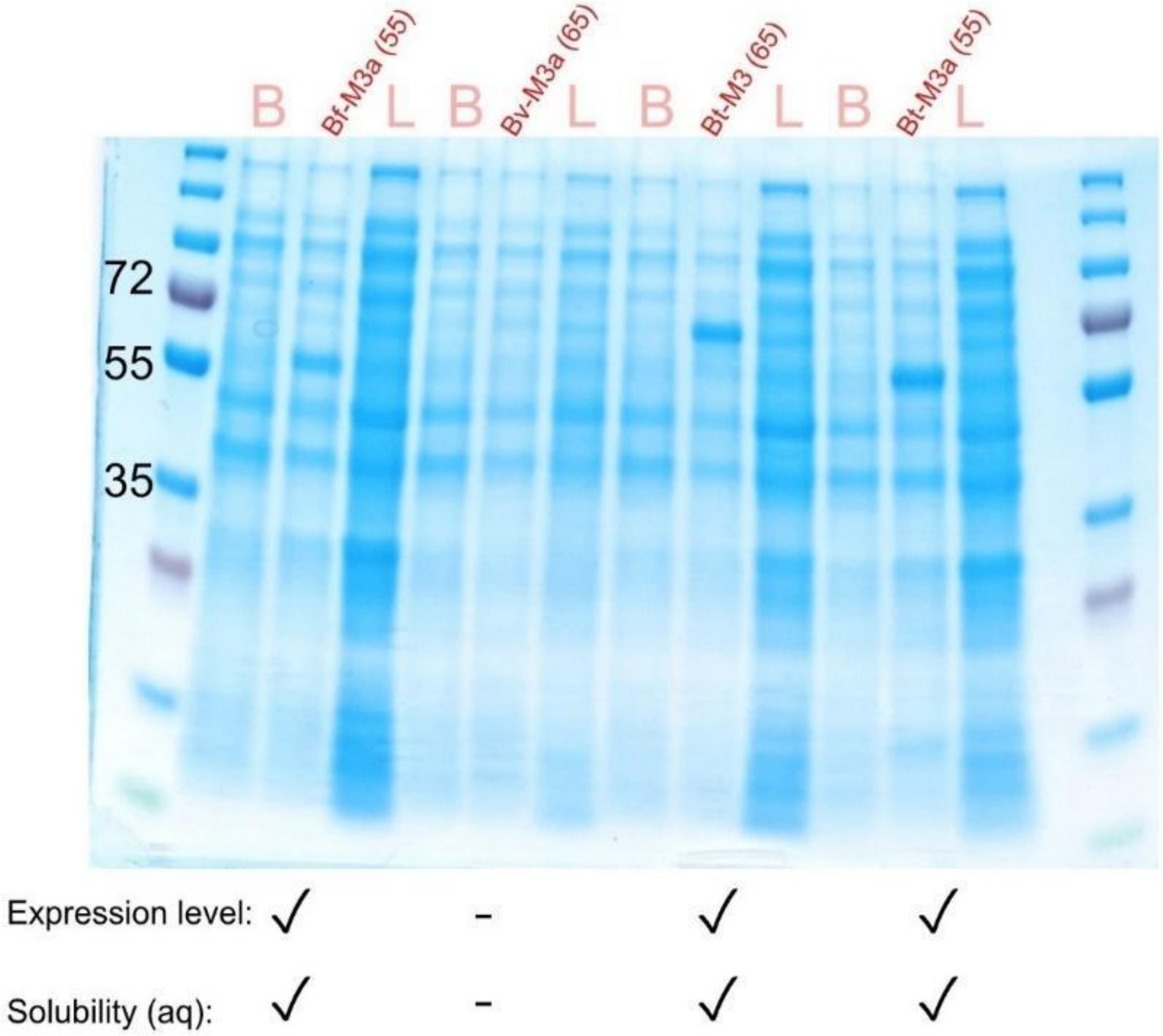

Figure S8. X-band EPR spectra recorded of cells expressing the *B. fragilis* group B [FeFe]-hydrogenase and empty vector BL21(DE3) control cells following anaerobic incubation with $[2Fe]^{pdt}$. A distinct partial rhombic EPR signal attributable to the H_{ox} state of the H-cluster with the first two g-values (g_1 = 2.101, g_2 = 2.053) observable in $[2Fe]^{pdt}$ -treated hydrogenase-expressing cells, while the third *g*-value is not discernible due to signal overlap with cell background (see also Supplementary Note 1). EPR spectra were recorded at 20 K, 64 μW microwave power, and at a microwave frequency of 9.36 GHz.

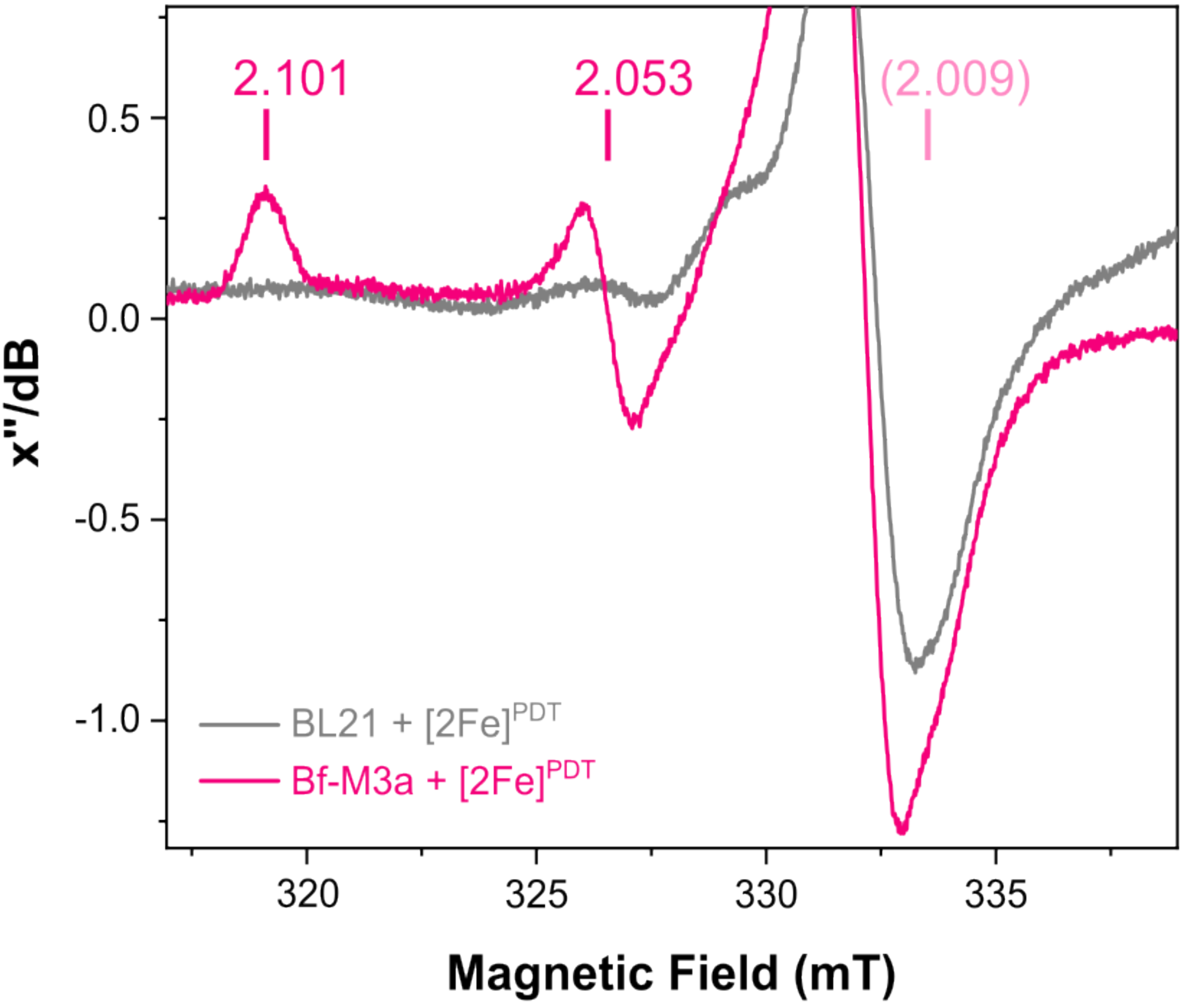

Figure S9. Growth (OD_{600}, green line, first right y axis), glucose consumption (mg/100ml, pink line, left y axis) and H_2 production (ppm, purple bars, second right y axis) observed for triplicate cultures of *Bacteroides fragilis* over 96 hours in mBMM media containing **a)** high and **b)** lower concentrations of glucose.

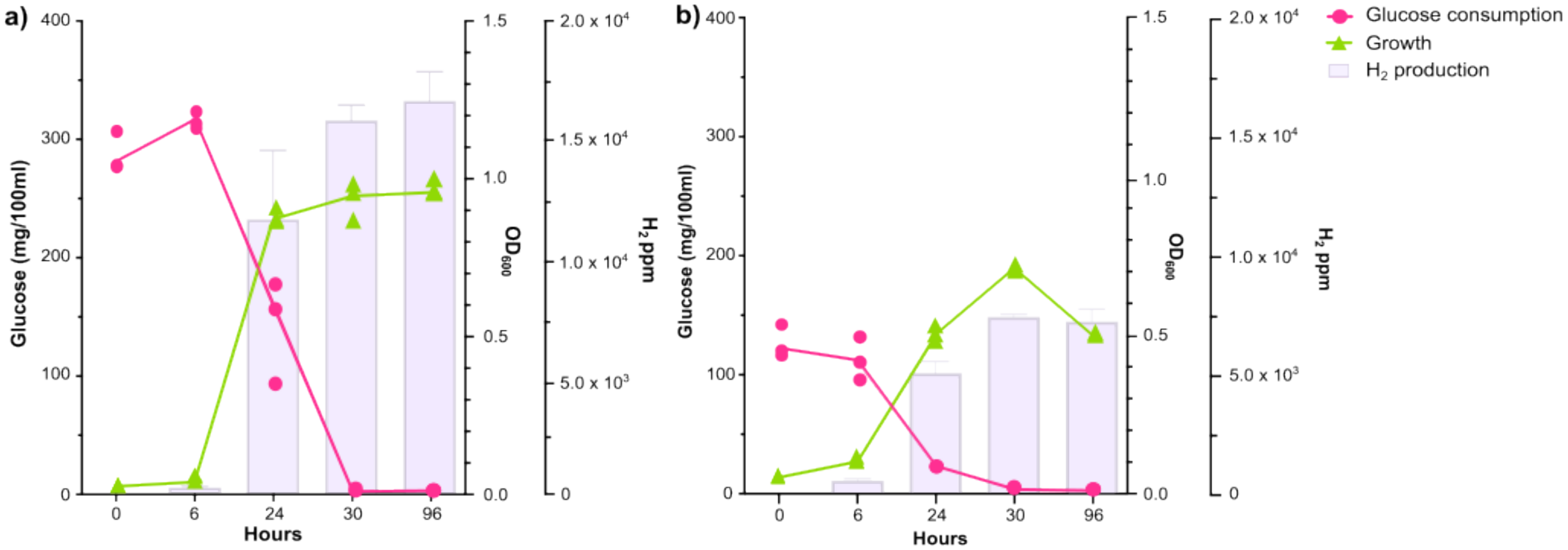
a)
b)
Glucose (mg/100ml)
OD600
H2 ppm
Hours
0
6
24
30
96
400
300
200
100
1.5
1.0
0.5
0.0
2.0 x 10^4
1.5 x 10^4
1.0 x 10^4
5.0 x 10^3
Glucose consumption
Growth
H2 production

Figure S10. Volatile fatty acid (VFA) measurements (uM) relative to growth of *B. fragilis* culture supernatant under the presence (+hemin, pink) or absence (-hemin, purple) of hemin (n = 3 replicates). Statistical significance was assessed using an unpaired t-test with Welch's correction, which does not assume equal variances between groups (*, P < 0.05; **, P < 0.01; ***, P < 0.001; ****, P < 0.0001). Error bars represent the standard deviation from the mean.

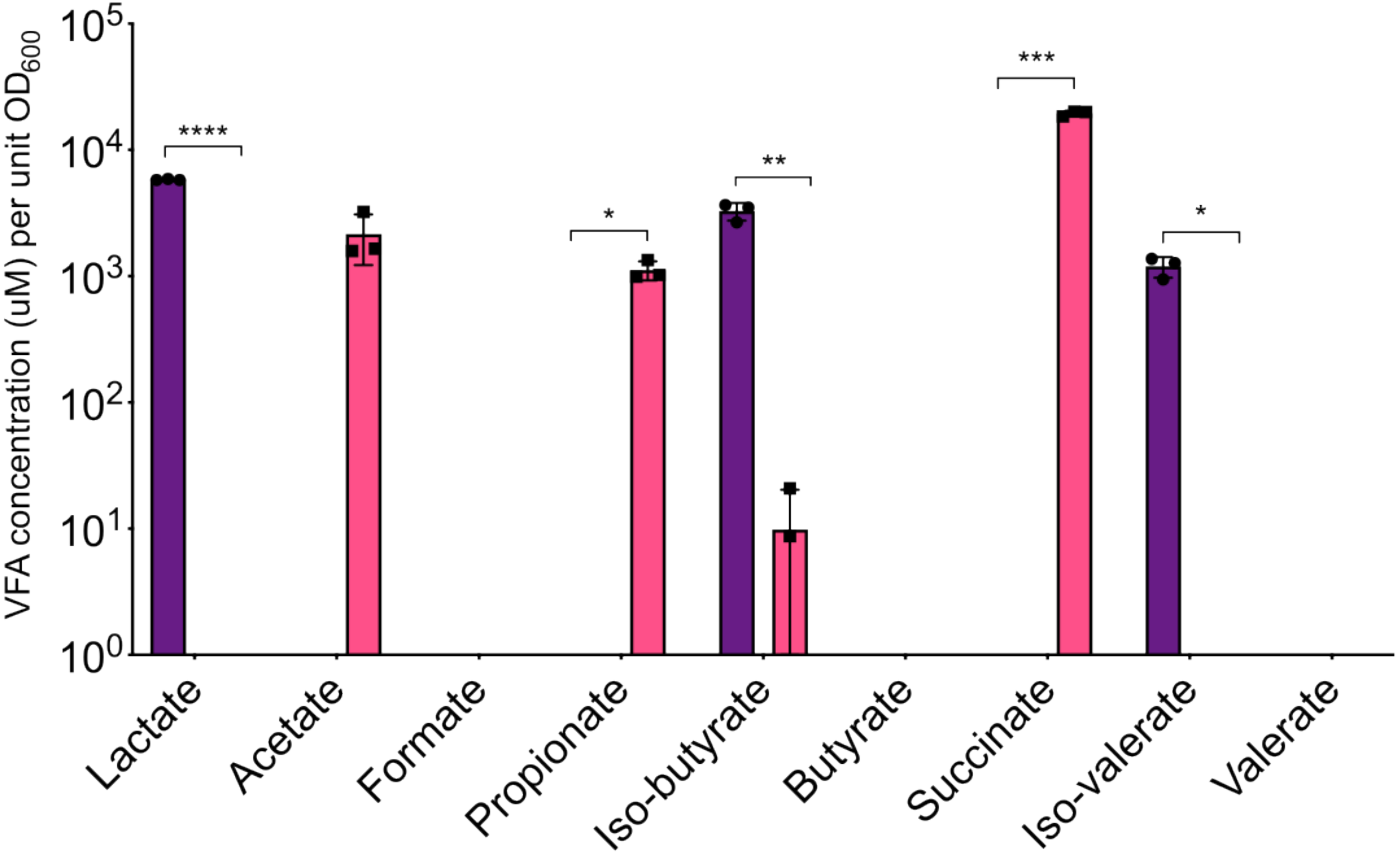

VFA concentration (uM) per unit OD_{600}
10^5
10^4
10^3
10^2
10^1
10^0
****
*
**
***
*
Lactate
Acetate
Formate
Propionate
Iso-butyrate
Butyrate
Succinate
Iso-valerate
Valerate

Figure S11. Differential fitness data of *B. thetaiotaomicron* random barcoded transposon mutant libraries exposed to various conditions. Data adapted from[1] (Fitness Browser https://fit.genomics.lbl.gov/cgi-bin/genesFit.cgi?orgId=Btheta&locusId=351362&locusId=349652&around=0). Statistically significant Group B [FeFe] hydrogenase transposon mutant fitness in different experimental conditions (different carbon sources, nitrogen sources, upon exposure to various stress conditions in rich brain heart infusion media (BHIS) or defined Varel and Bryant media (VBM)). Fitness values are represented as log2 ratios that indicate the change in abundance of mutants under each condition. A fitness score of 0 indicates mutants with an insertion in this gene did not confer a fitness defect, fitness score of < 0 means the gene was important for fitness under this condition, fitness of > 0 means that the loss of this gene gave mutants a growth advantage. Fitness score of ±1 is indicated on each graph as this score indicates that these mutants were 50% more or less abundant after exposure. Fitness scores between ±1 indicate subtle phenotypes, whereas fitness scores less than -2 or greater than 2 are considered strong phenotypes. All results shown are statistically significant with a reported absolute t-score of ≥4, a threshold adopted by the original authors[1].

1 Liu, H. *et al.* Functional genetics of human gut commensal Bacteroides thetaiotaomicron reveals metabolic requirements for growth across environments. *Cell reports* **34** (2021).

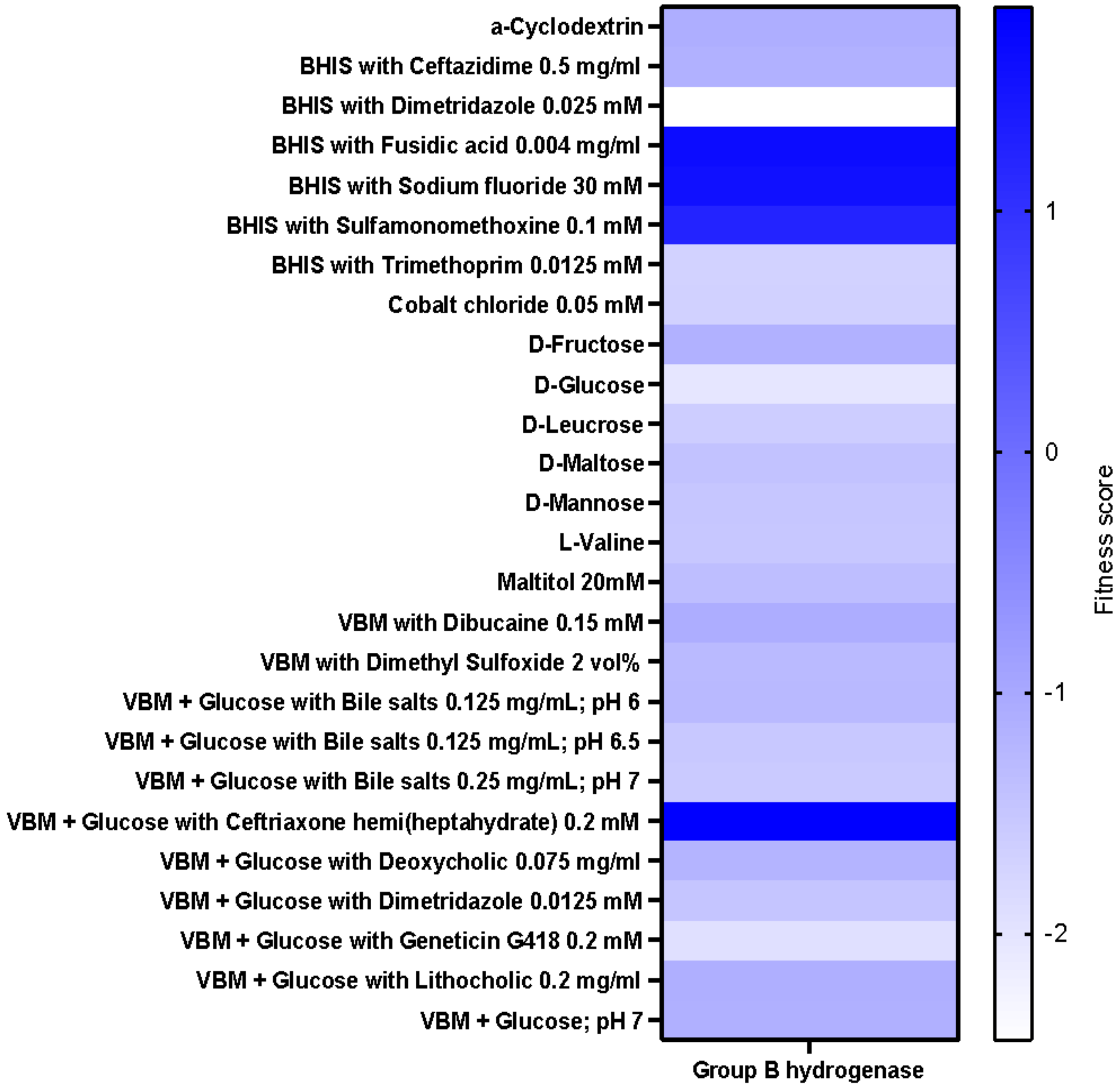

a-Cyclodextrin
BHIS with Ceftazidime 0.5 mg/ml
BHIS with Dimetridazole 0.025 mM
BHIS with Fusidic acid 0.004 mg/ml
BHIS with Sodium fluoride 30 mM
BHIS with Sulfamonomethoxine 0.1 mM
BHIS with Trimethoprim 0.0125 mM
Cobalt chloride 0.05 mM
D-Fructose
D-Glucose
D-Leucrose
D-Maltose
D-Mannose
L-Valine
Maltitol 20mM
VBM with Dibucaine 0.15 mM
VBM with Dimethyl Sulfoxide 2 vol%
VBM + Glucose with Bile salts 0.125 mg/mL; pH 6
VBM + Glucose with Bile salts 0.125 mg/mL; pH 6.5
VBM + Glucose with Bile salts 0.25 mg/mL; pH 7
VBM + Glucose with Ceftriaxone hemi(heptahydrate) 0.2 mM
VBM + Glucose with Deoxycholic 0.075 mg/ml
VBM + Glucose with Dimetridazole 0.0125 mM
VBM + Glucose with Geneticin G418 0.2 mM
VBM + Glucose with Lithocholic 0.2 mg/ml
VBM + Glucose; pH 7
Group B hydrogenase
1
0
-1
-2
Fitness score

Figure S12. Distribution of key [FeFe] hydrogenase subgroup genes across diseases. Statistical significance was assessed with Wilcoxon tests, using the Holm–Bonferroni method to account for multiple comparisons. IBD = inflammatory bowel disease. cpg = counts per genome. ME/CSF = myalgic encephalomyelitis / chronic fatigue syndrome.

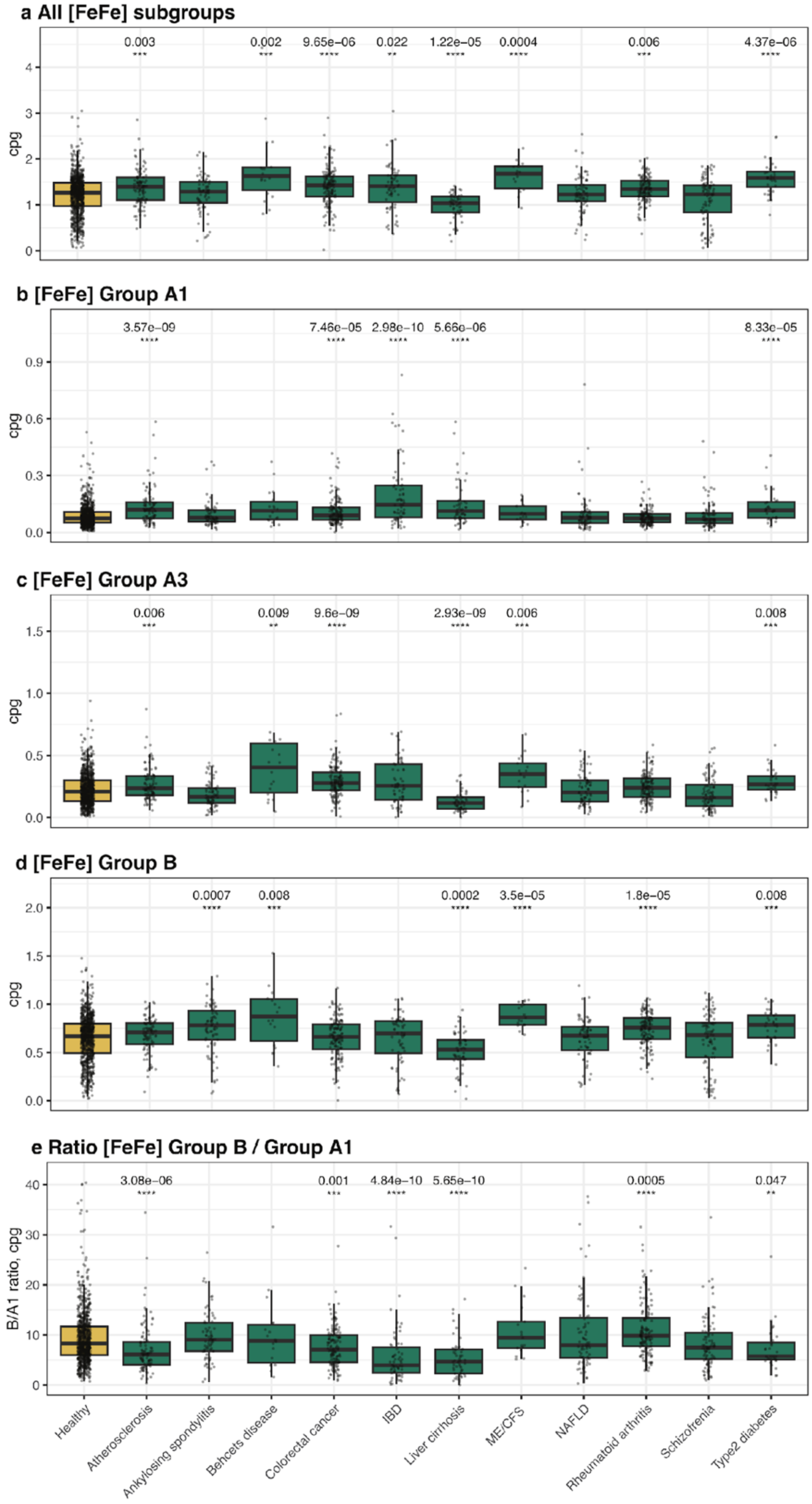

Figure S13. Distribution of key [NiFe] hydrogenase subgroup genes across diseases**.** Statistical significance was assessed with Wilcoxon tests, using the Holm–Bonferroni method to account for multiple comparisons. IBD = inflammatory bowel disease. cpg = counts per genome. ME/CSF = myalgic encephalomyelitis / chronic fatigue syndrome.

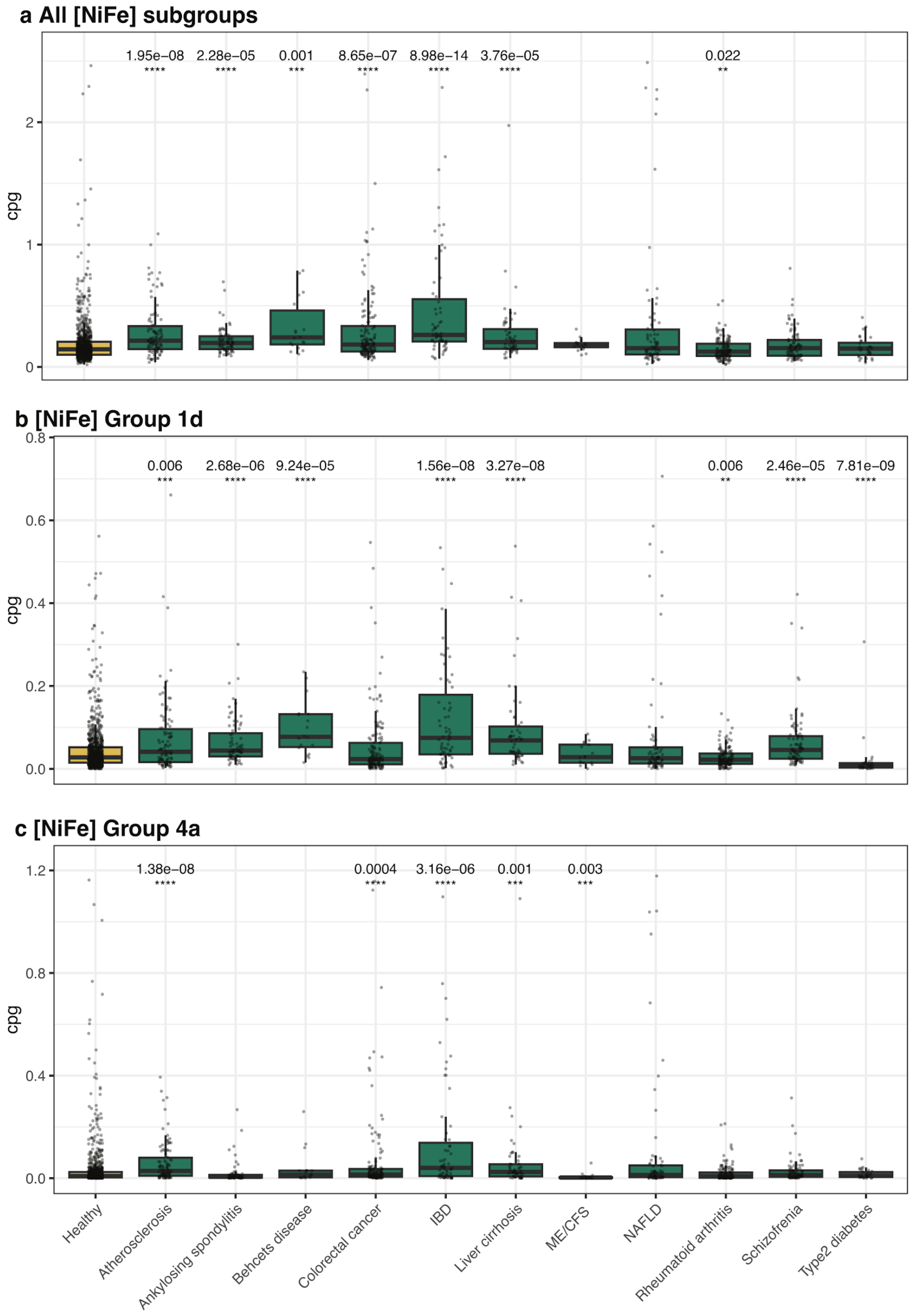

Supplement: Supplementary file 1 — Supplementary Notes 1–3, Dataset Legends 1–6, Tables 1–3 and Figs. 1–13. [file 41564_2025_2154_MOESM1_ESM.pdf]
