# Supplementary material for: A widespread hydrogenase supports fermentative growth of gut bacteria in healthy people
Source: Nat Microbiol. 2025 Oct 23;10(11):2686–701. doi: 10.1038/s41564-025-02154-w (PMC12578642; doi:10.1038/s41564-025-02154-w)
Supplement: Supplementary file 2 — Reporting Summary [file 41564_2025_2154_MOESM2_ESM.pdf]

nature portfolio

Corresponding author(s): Caitlin Welsh, Sam Forster, Chris Greening

Last updated by author(s): 08/08/2025

Reporting Summary

Nature Portfolio wishes to improve the reproducibility of the work that we publish. This form provides structure for consistency and transparency in reporting. For further information on Nature Portfolio policies, see our Editorial Policies and the Editorial Policy Checklist.

Statistics

For all statistical analyses, confirm that the following items are present in the figure legend, table legend, main text, or Methods section.

n/a	Confirmed	
☐	☒	The exact sample size (n) for each experimental group/condition, given as a discrete number and unit of measurement
☐	☒	A statement on whether measurements were taken from distinct samples or whether the same sample was measured repeatedly
☐	☒	The statistical test(s) used AND whether they are one- or two-sided<br>*Only common tests should be described solely by name; describe more complex techniques in the Methods section.*
☐	☒	A description of all covariates tested
☐	☒	A description of any assumptions or corrections, such as tests of normality and adjustment for multiple comparisons
☐	☒	A full description of the statistical parameters including central tendency (e.g. means) or other basic estimates (e.g. regression coefficient) AND variation (e.g. standard deviation) or associated estimates of uncertainty (e.g. confidence intervals)
☐	☒	For null hypothesis testing, the test statistic (e.g. F, t, r) with confidence intervals, effect sizes, degrees of freedom and P value noted<br>*Give P values as exact values whenever suitable.*
☒	☐	For Bayesian analysis, information on the choice of priors and Markov chain Monte Carlo settings
☒	☐	For hierarchical and complex designs, identification of the appropriate level for tests and full reporting of outcomes
☒	☐	Estimates of effect sizes (e.g. Cohen's d, Pearson's r), indicating how they were calculated

Our web collection on statistics for biologists contains articles on many of the points above.

Software and code

Policy information about availability of computer code

Data collection	Stool metagenome and metatranscriptome data was obtained from European Nucleotide Archives (v1.6.1) using 'enaBrowserTools' scripts that interface with the ENA servers, under accession number PRJNA389280
Data analysis	FastQC (v0.11.7) and MultiQC (v.1.0) were used for quality checking metagenome and metatranscriptome data<br>BBTools (v38.51) (specifically BBDuk) was used for trimming and filtering<br>SortmeRNA (v4.3.3) was used to remove rRNA sequences from metatranscriptomic reads<br>DIAMOND (v2.0) was used for homology-based searches of metagenomes, metatranscriptomes, isolate genomes and isolate transcripts against both the publicly available HydDB dataset and an in-house database (available at https://doi.org/10.26180/c.5230745).<br>Filtering and normalisation of homology-based search results was conducted as per methods presented in Lappan et al (2023, Nature Microbiology), Bay et al. (Nature Microbiology 2021,) and Ortiz et al (2021, PNAS) among others.<br>CheckM (v 1.1.3) was used for isolate genome quality checks<br>GTDB-Tk (v1.6.0) was used for taxonomic characterisation of gut isolates and phylogenetic analysis<br>Prokka (v1.14.6) was used for isolate genome annotation<br>Salmon (v1.9.0) was used for transcript quantification<br>DRAM (v1.4.6) was used for transcriptome annotation with the KEGG protein database<br>AlphaFold2 (v2.1.1) was used for structural modelling, along with Foldseek and ChimeraX (v1.6.1)<br>TrimGalore (v0.6.6) was used for disease cohort metagenome quality control<br>Bowtie (v2.3.552) was used to remove reads that mapped to the human genome from disease cohort analysis<br>Seqtk (v1.3) was used to normalise for sequence depth for disease cohort metagenomes.

For manuscripts utilizing custom algorithms or software that are central to the research but not yet described in published literature, software must be made available to editors and reviewers. We strongly encourage code deposition in a community repository (e.g. GitHub). See the Nature Portfolio guidelines for submitting code & software for further information.

Data

Policy information about availability of data

All manuscripts must include a data availability statement. This statement should provide the following information, where applicable:

- Accession codes, unique identifiers, or web links for publicly available datasets
- A description of any restrictions on data availability
- For clinical datasets or third party data, please ensure that the statement adheres to our policy

Stool metagenome and metatranscriptome datasets are available from ENA under accession number: PRJNA389280
Biopsy metagenome data is available from ENA under accession number: PRJEB45397
Isolate genomes are available from Australian Microbiome Culture Collection and ENA under accession numbers ERP105624 and ERP012217
Gut isolate genomes and transcripts are available from ENA under accession number: PRJEB70412

Research involving human participants, their data, or biological material

Policy information about studies with human participants or human data. See also policy information about sex, gender (identity/presentation), and sexual orientation and race, ethnicity and racism.

Reporting on sex and gender	54% of participants were female, primarily aged between 11-18 years of age. Considering this study was focused on microorganisms cultured from the biopsy samples, patient sex, gender and other demographics weren't analysed or controlled for.
Reporting on race, ethnicity, or other socially relevant groupings	Not applicable
Population characteristics	42 pediatric patients with non-inflammatory gastrointestinal conditions.
Recruitment	Patients were recruited during pediatric endoscopy lists at Monash Children's Hospital from consenting participants receiving clinically indicated colonoscopies.
Ethics oversight	Human Research Ethics Committee (HREC) (HREC/16/MonH/253) and Monash 435 University Ethics Committee (Monash Health ref. 16367A

Note that full information on the approval of the study protocol must also be provided in the manuscript.

Field-specific reporting

Please select the one below that is the best fit for your research. If you are not sure, read the appropriate sections before making your selection.

☒ Life sciences ☐ Behavioural & social sciences ☐ Ecological, evolutionary & environmental sciences

For a reference copy of the document with all sections, see nature.com/documents/nr-reporting-summary-flat.pdf

Life sciences study design

All studies must disclose on these points even when the disclosure is negative.

Sample size	Sample size was not statistically predetermined and co-variates were not controlled for. The sample size of metagenomes, metatranscriptomes and isolate genomes was based on available, relevant datasets and what was considered representative of gastrointestinal microbiota. The sample size of cultured isolates was chosen based on ensuring taxonomic diversity, as well as including only those isolates that encoded the genes of interest.
Data exclusions	Six isolate genomes were excluded from analysis due to having high contamination (>10%) reported via CheckM.
Replication	Biological triplicates were used throughout the study for all experimental work, and all replication was successful.
Randomization	As this was a study involving genomic, biochemical and physiological analysis of microorganisms, randomisation was not necessary.
Blinding	As this was a study involving genomic, biochemical and physiological analysis of microorganisms, blinding was not necessary.

Reporting for specific materials, systems and methods

We require information from authors about some types of materials, experimental systems and methods used in many studies. Here, indicate whether each material, system or method listed is relevant to your study. If you are not sure if a list item applies to your research, read the appropriate section before selecting a response.

Materials & experimental systems

n/a	Involved in the study
☒	☐ Antibodies
☒	☐ Eukaryotic cell lines
☒	☐ Palaeontology and archaeology
☒	☐ Animals and other organisms
☒	☐ Clinical data
☒	☐ Dual use research of concern
☒	☐ Plants

Methods

n/a	Involved in the study
☒	☐ ChIP-seq
☒	☐ Flow cytometry
☒	☐ MRI-based neuroimaging

Plants

Seed stocks	*Report on the source of all seed stocks or other plant material used. If applicable, state the seed stock centre and catalogue number. If plant specimens were collected from the field, describe the collection location, date and sampling procedures.*
Novel plant genotypes	*Describe the methods by which all novel plant genotypes were produced. This includes those generated by transgenic approaches, gene editing, chemical/radiation-based mutagenesis and hybridization. For transgenic lines, describe the transformation method, the number of independent lines analyzed and the generation upon which experiments were performed. For gene-edited lines, describe the editor used, the endogenous sequence targeted for editing, the targeting guide RNA sequence (if applicable) and how the editor was applied.*
Authentication	*Describe any authentication procedures for each seed stock used or novel genotype generated. Describe any experiments used to assess the effect of a mutation and, where applicable, how potential secondary effects (e.g. second site T-DNA insertions, mosiacism, off-target gene editing) were examined.*
